# Epidemiological analysis of hemorrhagic fever with renal syndrome in China with the seasonal-trend decomposition method and the exponential smoothing model

**DOI:** 10.1038/srep39350

**Published:** 2016-12-15

**Authors:** Guibao Ke, Yao Hu, Xin Huang, Xuan Peng, Min Lei, Chaoli Huang, Li Gu, Ping Xian, Dehua Yang

**Affiliations:** 1 Department of Nephrology, Affiliated Hospital/Clinical Medical College of Chengdu University, Chengdu, People’s Republic of China; 2 Administration office, Affiliated Hospital/Clinical Medical College of Chengdu University, Chengdu, People’s Republic of China

## Abstract

Hemorrhagic fever with renal syndrome (HFRS) is one of the most common infectious diseases globally. With the most reported cases in the world, the epidemic characteristics are still remained unclear in China. This paper utilized the seasonal-trend decomposition (STL) method to analyze the periodicity and seasonality of the HFRS data, and used the exponential smoothing model (ETS) model to predict incidence cases from July to December 2016 by using the data from January 2006 to June 2016. Analytic results demonstrated a favorable trend of HFRS in China, and with obvious periodicity and seasonality, the peak of the annual reported cases in winter concentrated on November to January of the following year, and reported in May and June also constituted another peak in summer. Eventually, the ETS (M, N and A) model was adopted for fitting and forecasting, and the fitting results indicated high accuracy (Mean absolute percentage error (MAPE) = 13.12%). The forecasting results also demonstrated a gradual decreasing trend from July to December 2016, suggesting that control measures for hemorrhagic fever were effective in China. The STL model could be well performed in the seasonal analysis of HFRS in China, and ETS could be effectively used in the time series analysis of HFRS in China.

Hemorrhagic fever with renal syndrome (HFRS, also known as epidemic hemorrhagic fever), which is also referred to as epidemic hemorrhagic fever, is a kind of natural focal disease that is induced by Hantaan virus, and carried and transmitted by rodents, which are the natural reservoir for hantaviruses^1^,^2^. Symptoms of HFRS usually develop within 1 to 2 weeks after exposure to infectious material, initial symptoms include intense headaches, back and abdominal pain, fever, chills, nausea, and blurred vision. Later symptoms can include low blood pressure, acute shock, vascular leakage, and acute kidney failure, which can cause severe fluid overload^3^. Active and effective monitoring is one of the most effective measures for controlling the HFRS epidemic. As the country with the most reported HFRS cases in the world, the epidemic in China has always been one of the most important problem, in the recent 30 years, the efforts to control HFRS in China have been increased, and great achievements have been attained in vaccine development, but the threat of HFRS epidemic has not been completely eliminated yet. A research by Zhang^4^ *et al*. forecasted the variation trend before 2004, which indicated a potential prevalence.

The epidemic of numerous infectious diseases is associated with the variation characteristics of periodicity and seasonality^5^,^6^,^7^. Similar to other diseases, the epidemiological characteristics of the time-based characteristics of HFRS have been one of the focused issues for long-term attention. The epidemic of HFRS shows certain periodicity, and lots of cases can be seen during the peak period, while sporadic cases can be observed during the non-peak period. In China, scholars analysed the annual report data of HFRS in China through the time series model^8^; and some characteristics could be found from the annual data; however, there were still some unclear problems regarding some trends within the year, such as the specific variation trend of each year. The National Health and Family Planning Commission of China (NHFPC, originally known as the Chinese Ministry of Health) has promulgated a National HFRS monitoring program (Trial) in 2005^9^, focusing particularly on measuring the public health intervention’s effectiveness on HFRS control, with the implementation of this policy. Currently, little literature analyses the variation characteristics of HFRS within a year or determines the variation characteristics in recent years, as well as the periodical variation within the yearly data through the monthly data, and the determination of these conditions is of essential importance to the seasonal distribution of the control resources every year. In order to further determine these questions, we adopted the Seasonal-trend decomposition (STL) and exponential smoothing model (ETS) methods to analyse the monthly data from the National Heath and Family Planning Commission Reports, and analysed some specific conditions of the periodicity and seasonality of the monthly data.

## Materials and Methods

### Data resource

The reported HFRS data from January 2006 to June 2016 was derived at August 25 and 26, 2016, from the National Heath and Family Planning Commission (http://www.nhfpc.gov.cn/), and the same data could also be seen in the Chinese Center for Disease Control [ http://www.chinacdc.cn/], and they were assembled as monthly counts of the reported cases.

### Statistical Analysis

#### STL analysis

One of the most challenges in data analysis of time series is the selection of an adequate model to describe seasonal components, in this paper, the Seasonal-Trend Decomposition based on locally-weighted regression (Loess) known as STL, which was originally presented by Cleveland in 1990, was selected as a filtering procedure designed for decomposing a time series into trend, seasonal, and remainder components^10^:


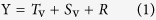


where *Y*_*v*_ is the component of original time series, *T*_*v*_ is the component of trend variation that can be viewed as change tendency with low frequency, *S*_*v*_ is the component seasonal variation that can be regarded as variations with high frequency due to stable seasonal disturbance, and *R* is the component of remainder variation that can be viewed as irregular variation due to random disturbance. STL works as an iterative nonparametric regression procedure using a series of LOESS smoothers, which is based on fitting a weighted polynomial regression. In detail, LOESS produces a smoothed estimate (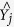) that is defined by the following:


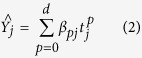


where *β*_*pj*_ is the (*d* + 1)-dimensional least squares estimate of the weighted regression, 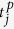 is the (*d* + 1)-dimensional vector of the time of observation, j is the number of time lags up to the maximum defined by the smoothing parameter (*n*), *p* = 0, …, *d*, and *d* is the degree of the polynomial fitting^11^. Finally, the estimates of both components are then used to compute the remainder: *R* = *Y *− *T*_v_ − *S*_v_^12^. With the above-mentioned procedure, the STL can in turn detect both the overall and seasonal variation of a time series.

In this paper, seasonal time trends for HFRS was analysed using the STL method via the stl() function in R software, which enables each of the components to be isolated and analysed, according to Hyndman1’s definition in R^13^,^14^, two main parameters (the trend window (t.window) and seasonal window (s.window) can control how rapidly the trend and seasonal components can change.

#### ETS model

The exponential smoothing model (ETS) method is a kind of forecasting method which takes the historical information into comprehensive consideration; with weighting observed values, the forecasting value can comprehensively reflect all the historical information, and take the effect of time variation on the forecasting value into consideration^15^,^16^. ETS model considers an original time series as a combination of the trend (T), seasonal (S) and error (E) components, which can be additive (A), multiplicative (M) or none (N). There ETS method contains several methods in detail, such as single exponential smoothing, double exponential smoothing, Holt trend exponential smoothing (with or without seasonal characteristics), and some other methods based on the various characteristics of the original series. According to Yang’s description, the trend components consists of another combination of a level term (*l*) and a growth term (*b*). the forecast trend *T*_*h*_ over the next h time periods, (*l*) and b can be combined in the following 5 ways:


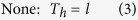



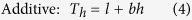



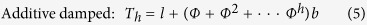



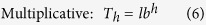



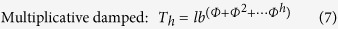


where 0 < *Φ* < 1 is defined as the damping parameter, and the seasonal components can be additive(A), multiplicative(M) or none(N). When it comes to the seasonal components, it can be additive (*T* × *S*), multiplicative (*T* × *S*) or none. This gives rise to the combinations of time series components as shown in Table 1:

In E-views, parameters like A, N and M were automatically selected through setting the automatic selection mode, the optimal model from the 30 candidate models for fitting and forecasting; the model selection is conducted with the minimal Bayesian Information Criterion (BIC) principle, a residual test was then performed with the Ljung-Box Q test; in the meantime, MAPE is also utilized to test the accuracy (3):


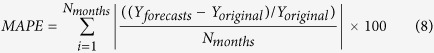


According to Lee^17^, the MAPE of less than or equal to 10% means highly accurate forecasts, 10% < MAPE < 20% means good forecasts; 20% < MAPE < 50% means reasonable forecasts, and MAPE > 50% suggests inaccurate forecasting.

In the statistic E-views which was designed well for time-series analysis, provides was designed as a built-in analytic procedure (the exactly analytic procedure of ETS in E-views 8 can be seen from the paper “***ETS Exponential Smoothing in EViews 8**”* in the official website of E-views: http://www.eviews.com/EViews8/ev8ecets_n.html)^18^.

The analysis in this research adopted R and Econometric Views 8 (E-views 8) (E-Views is a statistical package is developed by Quantitative Micro Software (QMS), and mainly designed for time-series analysis, it is currently a more and more popular program that widely used in time series modeling in various fields for the fitting and forecasting analysis of the HFRS data, with *α* = 0.05 being the significant level.

This paper has been approved by Affiliated Hospital/Clinical Medical College of Chengdu University, as aggregated data with no personal information were involved in this study.

## Results

### General information

We summarized the monthly reported cases in each year to analyze the overall annual variation trend from 2006 to 2015 in China, the results of which revealed that HFRS in China has been continuously from 2006 to 2015, and the variation trend could be divided into three sections; incidence cases remarkably decreased from 16129 to 9203 from 2006 to 2009, with the percent change of −42.94%; it rose to 13918 to year 2012, with the percent change of 51.23% relative to that in 2009; and with a following decrease from 2012 to 2016, with the percent change of 61.64%. On the whole, decreasing trend was observed from 2006 to 2015, with the percent change of −66.90%; Since 2005, the National Health and Family Planning Commission of China has promulgated a National HFRS monitoring program (Trial)^9^, focusing particularly on measuring the public health intervention’s effectiveness on HFRS control, descriptive analysis of HFRS incidence from 2006 to 2015 indicated that the prevention control has attained certain achievements as the disease incidence continuously declined during this period.

**Figure 1:**
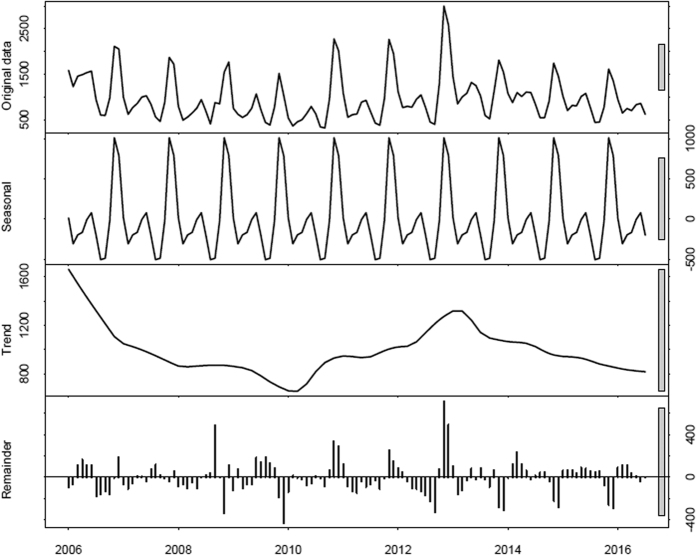
The disintegrating results of HFRS by the STL method. It could be seen obviously that the variation of HFRS in China showed the periodicity with one year (12 months) being a cycle; there was obvious seasonality during the variation process in each year; and there were two peaks in each cycle. From the seasonal angle, the series showed a 12-month stochastic seasonality in the reporting pattern of HFRS, From the trend angle, we can see a downward overall trend and periodically change of disease incidence; From the reminder angle, we can also see a 12-month stochastic variance.

**Figure 2:**
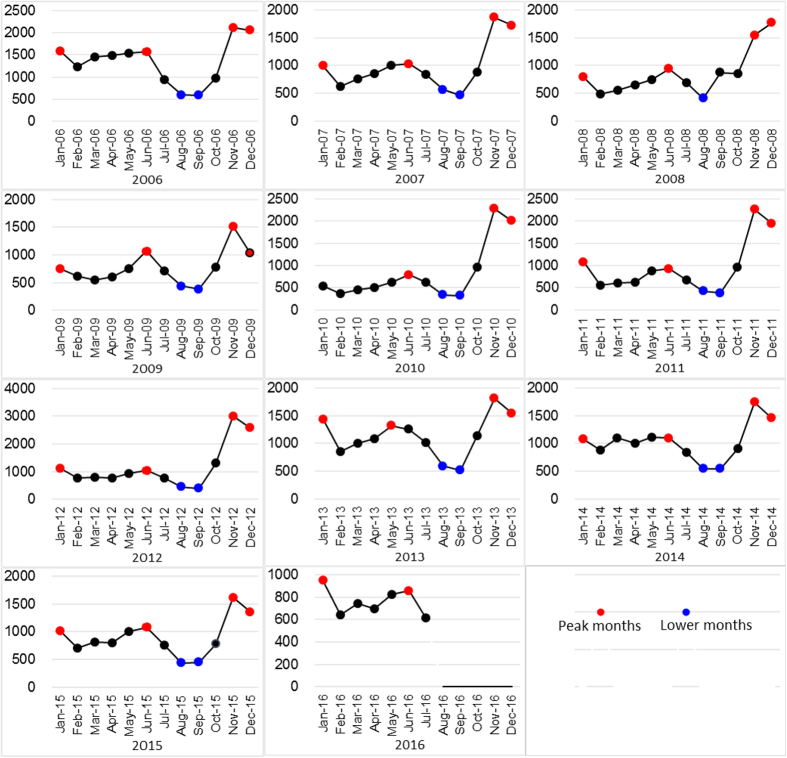
The precise variation of the reported HFRS cases in each year after being disintegrated according to different years, among which the red points represented the months with relatively more reported cases in each year, which mainly concentrated on November, December, and January; while the blue points represented the months with relatively few reported cases, which were August and September.

Decomposed the monthly data of HFRS into the overall trend and the seasonal trend through the STL analysis, we can isolate seasonality and trend components from the monthly HFRS data series and also eliminate part of the random noise or reminder component. As shown in Fig. 1, the variability of each component separately over the timescale. From the seasonal trend, the series showed a 12-month stochastic seasonality in the reporting pattern of HFRS, From the trend trend, we can see a downward overall trend and periodically change of disease incidence; From the reminder angle, we can also see a 12-month stochastic variance; Fig. 2 described the data variation in each year after being decomposed, it could be seen from the analysis results that the monthly data of HFRS had the year-based periodicity, the data in each year had distinct periodicity and seasonality, there were 2 peaks of the reported cases, the epidemic showed 2 peaks, which were summer and winter, and the reported cases in winter were higher than those in summer. May and June in summer would witness the first peak of the reported cases, and the most reported cases each year mainly concentrated on November to January in the following year, and August and September had the least reported cases.

**Figure 3:**
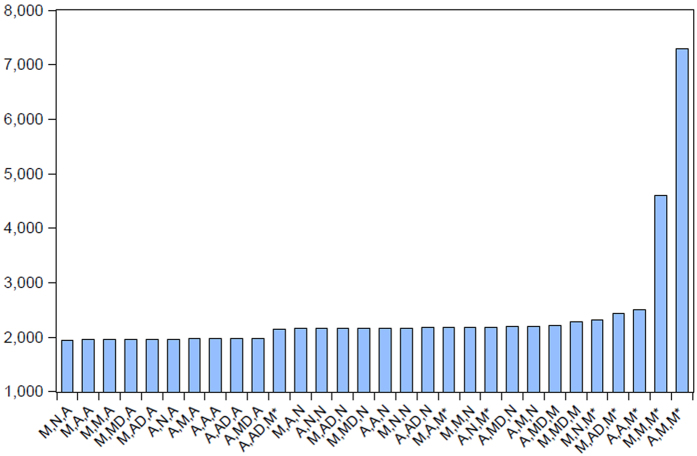
BIC comparison of adopting the ETS model to forecast HFRS. The model selection is conducted with the minimal Bayesian Information Criterion (BIC) principle, and ETS (M, N and A) (BIC = 1946.14, see Table 2 and Fig. 3) was selected to be the optimal model for fitting and forecasting. (**Note**: A: Additive, N: None, M: Multiplicative, Ad: Additive damped, Md: Multiplicative damped).

**Figure 4:**
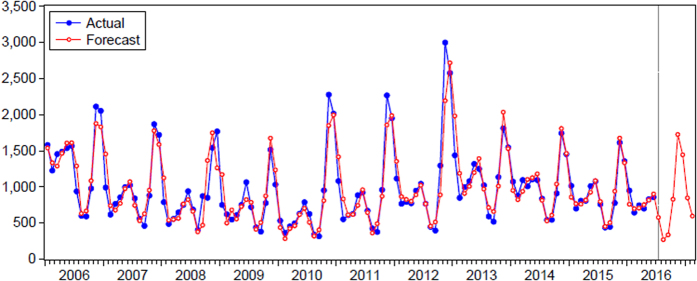
Fitting and forecasting results of the reported HFRS cases by ETS (M, N, and A).

The ETS model was run by the E-views software, and altogether 30 candidate models were enrolled in the analysis as the candidate models through the various combinations of the single parameters like A, N and M. Refer to for the fitting and forecasting of the monthly data of HFRS. ETS (M, N and A) (BIC = 1946.14, see Table 2 and Fig. 3) was determined to be the optimal model for fitting and forecasting (refer to Fig. 4 for the fitting and forecasting results) under the minimal BIC principle, and forecasts of incidence cases from July to December 2016 were: 577, 268, 334, 827, 1725, 1444. Ljung-Box Q test indicated that ETS (M, N, and A) was closer to achieve white noise (*P*_Box-Ljung_ > 0.05); the goodness test of fit, which demonstrated that MAPE = 13.12%, suggested that the model had good fitting according to the judgment criteria of Lee *et al*.^17^.

## Discussion

HFRS is a kind of highly fatal infectious disease with murine being the major source of infection, and HFRS has caused severe influence worldwide^19^. HFRS has milder epidemic situation in Europe and America, and it mostly distributes in Asian countries, among which China is the country that are mostly affected, and HFRS cases can be seen in most areas^20^. The incidence of HFRS is highly variable at the states level. Our results clearly show that the HFRS incidence in China have decreased dramatically in during the last decade, which is similar to the general trend in several countries in Asia, as far back as 1920s to 1930s, China witnessed the prevalence of HFRS. The targeted vaccine development has lasted for decades since the isolation of the Hantaan virus at home and abroad in 1980 successively; after almost 20 years of efforts, multiple HFRS-targeted vaccines which have played an important role in the control of HFRS in China, have been developed^21^,^22^. Currently, the prevention and treatment of HFRS in China follows the principle of “three-early and one-in-place”, namely, early discovery, early rest, early treatment and in-place isolation treatment, and it renders great progress in the prevention of HFRS, but it is still faced with challenges, with the implementation of NHFPC’s National HFRS monitoring program (Trial) since 2005, the disease incidence detected in most provinces showed significant decrease as in some previous studies^23^,^24^. The analytic result in this paper, which showed that a decreasing trend was obtained from 2006 to 2015, also indicated that the epidemic trend of HFRS in China was under control, and the prevention and control had attained certain achievements. Even though, the annual reported HFRS cases in China remains the top in the world, which is higher than that in American and European countries^25^; With the utilization of the time series model, the short term predicting result over the next year is expected that HFRS incidence will continue to decline, implies that the national monitoring program will continue to operate effectively in HFRS control in the near future.

Obtaining the original data series through the reported data, and analyzing the spatial-temporal characteristics through the time series analysis method is an important method to analyze the time data in the epidemiology, which can effectively obtain the important characteristics of data variation, such as periodicity and seasonality^22^; in addition, the short-term and long-term forecast can evaluate the control measures, in the meantime, it can adopts effective and timely solutions for the epidemic peak that may occur or the reappeared prevalence or outbreak^26^. Some scholars analyze the annual reported data of HFRS in China through time series model like ARMA, analyze the variation trends, seasonal trends and epidemic characteristics of the annual data of HFRS, and verify the effectiveness of the model^27^,^28^. In this research, we analyze the monthly data variation characteristics of HFRS in China only through the STL method. We determine the periodicity of the annual variation of incidence cases, and further determine the concern of the variation in each year through disintegrating the annual data. The epidemic shows two peaks of the reported cases, which are summer and winter, and the cases reported in winter are higher than those in summer. Figure 2 describes the data variation each year after disintegration, May and June in summer will witness the first peak of the reported cases, and the most reported cases each year mainly concentrate on November to January in the following year, and August and September have the least reported cases. Therefore, the relevant departments should conduct corresponding resource allocation for the months of peaks and those with few reported cases according to the incidence, when they are formulating the control policies for HFRS. We forecast the incidence from June to December 2016 through analyzing the ETS model, the results of which suggest that the incidence of HFRS in China still shows periodicity, but the overall condition shows a gradual decreasing trend. Meanwhile, it can be discovered from the model test results that ETS model has good fitting and forecasting accuracy for the incidence of HFRS, and it is suitable for monitoring the morbidity; therefore, we suggest that the time series models, such as ETS be adopted appropriately in the subsequent epidemic research on HFRS, so as to help decision-making.

## Additional Information

**How to cite this article**: Ke, G. *et al*. Epidemiological analysis of hemorrhagic fever with renal syndrome in China with the seasonal-trend decomposition method and the exponential smoothing model. *Sci. Rep.* **6**, 39350; doi: 10.1038/srep39350 (2016).

**Publisher’s note:** Springer Nature remains neutral with regard to jurisdictional claims in published maps and institutional affiliations.

## Figures and Tables

**Table 1:** Definitions of A, N, M and D in the ETS (A, N, M) model.

Trend	Seasonal		
	N (None)	A (Additive)	M (Multiplicative)
N (None)	N, N	N, A	N, M
A (Additive)	A, N	A, A	A, M
A_d_ (Additive damped)	A_d_, N	A_d_, A	A_d_, M
M (Multiplicative)	M, N	M, A	M, M
M_d_ (Multiplicative damped)	M_d_, N	M_d_, A	M_d_, M

**Table 2:** Characteristics of 30 candicate models.

Model	Compact LL	Likelihood	AIC	BIC*	HQ	AMSE
ETS(M,N,A)	**−939.214**	**−813.315**	**1906.43**	**1946.14**	**1922.56**	**82525.8**
ETS(M,A,A)	**−**937.917	**−**812.018	1907.83	1953.22	1926.27	83565.8
ETS(M,M,A)	**−**939.194	**−**813.294	1910.39	1955.77	1928.82	82867.9
ETS(M,MD,A)	**−**939.214	**−**813.315	1912.43	1960.64	1932.02	82525.8
ETS(M,AD,A)	**−**939.214	**−**813.315	1912.43	1960.64	1932.02	NA
ETS(A,N,A)	**−**950.251	**−**824.351	1928.50	1968.21	1944.63	75637.7
ETS(A,M,A)	**−**949.251	**−**823.351	1930.50	1975.88	1948.94	73936.9
ETS(A,A,A)	**−**950.163	**−**824.264	1932.33	1977.71	1950.76	75562.4
ETS(A,AD,A)	**−**949.861	**−**823.961	1933.72	1981.94	1953.31	NA
ETS(A,MD,A)	**−**950.251	**−**824.351	1934.50	1982.72	1954.09	75637.7
ETS(A,AD,M*)	**−**1036.54	**−**910.640	2107.08	2155.30	2126.67	NA
ETS(M,A,N)	**−**1069.72	**−**943.825	2147.45	2158.79	2152.06	556678
ETS(A,N,N)	**−**1075.91	**−**950.014	2155.83	2161.50	2158.13	466407
ETS(M,AD,N)	**−**1069.72	**−**943.825	2149.45	2163.63	2155.21	556678
ETS(M,MD,N)	**−**1073.48	**−**947.583	2156.97	2171.15	2162.73	778342
ETS(A,A,N)	**−**1075.90	**−**950.004	2159.81	2171.15	2164.42	466278
ETS(M,N,N)	**−**1081.75	**−**955.853	2167.50	2173.18	2169.81	466363
ETS(A,AD,N)	**−**1075.91	**−**950.014	2161.83	2176.01	2167.59	NA
ETS(M,A,M*)	**−**1049.80	**−**923.898	2131.60	2176.98	2150.03	6.1E + 08
ETS(M,M,N)	**−**1079.67	**−**953.772	2167.34	2178.69	2171.95	649141
ETS(A,N,M*)	**−**1057.33	**−**931.431	2142.66	2182.37	2158.79	182090
ETS(A,MD,N)	**−**1084.20	**−**958.298	2178.40	2192.58	2184.16	236265
ETS(A,M,N)	**−**1086.83	**−**960.928	2181.66	2193.00	2186.26	260907
ETS(A,MD,M)	**−**1067.79	**−**941.887	2169.57	2217.79	2189.16	4223849
ETS(M,MD,M)	**−**1102.81	**−**976.912	2239.62	2287.84	2259.21	9103897
ETS(M,N,M*)	**−**1122.26	**−**996.356	2272.51	2312.22	2288.64	1.9E + 07
ETS(M,AD,M*)	**−**1174.46	**−**1048.57	2382.93	2431.15	2402.52	NA
ETS(A,A,M*)	**−**1213.46	**−**1087.56	2458.92	2504.30	2477.36	2467098
ETS(M,M,M*)	**−**2260.91	**−**2135.01	4553.83	4599.21	4572.27	6.0E + 23
ETS(A,M,M*)	**−**3612.79	**−**3486.89	7257.59	7302.97	7276.03	7.7E + 23

^*^NA: failed to converge.
